# Bridging the Knowledge Gap in Harmaline’s Pharmacological Properties: A Focus on Thermodynamics and Kinetics

**DOI:** 10.3390/pharmaceutics18010035

**Published:** 2025-12-26

**Authors:** Tatyana Volkova, Olga Simonova, German Perlovich

**Affiliations:** G.A. Krestov Institute of Solution Chemistry RAS, 153045 Ivanovo, Russia

**Keywords:** harmaline, solubility thermodynamics, distribution thermodynamics, permeability, kinetic study

## Abstract

**Background/Objectives**: Advancing information on the key physicochemical properties of biologically active substances enables the development of formulations with reduced dosing, lower toxicity, and minimal adverse effects. This work addresses the knowledge gap concerning the pharmacologically relevant properties of harmaline (HML), with a focus on thermodynamic and kinetic aspects. New data were obtained on the compound’s solubility and distribution coefficients across a wide temperature range. Specifically, solubility was measured in aqueous buffers (pH 2.0 and 7.4), 1-octanol (OctOH), *n*-hexane (Hex), and isopropyl myristate (IPM), while distribution coefficients were determined in OctOH/pH 7.4, Hex/pH 7.4, and IPM/pH 7.4 systems. **Methods**: Three membranes—regenerated cellulose (RC), PermeaPad (PP) and polydimethylsiloxane-polycarbonate (PDS)—were used as barriers in permeability studies using a Franz diffusion cell. **Results**: At 310.15 K, the molar solubility of HML in the solvents decreased in the following order: OctOH > pH 2.0 > pH 7.4 > IPM > Hex. The distribution coefficient of HML showed a strong dependence on the nature of the organic phase, correlating with its solubility in the respective solvents. The OctOH/pH 7.4 distribution coefficient ranged from 0.973 at 293.15 K to 1.345 at 313.15 K, falling within the optimal range for potential drug bioavailability. The transfer of HML into OctOH (from either pH 7.4 or hexane) is thermodynamically spontaneous, whereas its transfer into Hex is unfavorable. **Conclusions**: Based on its permeability across the PP barrier, HML was classified as highly permeable. The distribution and permeation profiles of HML showed similar trends over 5 h in both the OctOH/pH 7.4–PP and IPM/pH 7.4–PDS systems. These systems were therefore proposed as suitable models for studying HML transport in vitro.

## 1. Introduction

Common harmal (*Peganum harmala*) has been used in folk medicine since ancient times. Both harmine and harmaline belong to the harmala alkaloid beta-carboline group. Harmine is a beta-carboline alkaloid that is predominant in the roots of the plant, while harmaline (HML) is a psychoactive alkaloid more common in the seeds. HML is a reversible inhibitor of monoamine oxidase (MAO) and a central nervous system stimulant. It has been used for thousands of years, along with related alkaloids, in traditional medicine across Asia, the Middle East, and Amazonia. Its diverse pharmacological profile includes demonstrated inhibitory activity against multiple cancer cell lines [1], the ability to decrease systemic arterial blood pressure and total peripheral vascular resistance [2], and a neuroprotective effect [3,4]. Furthermore, its strong inhibition of MAO underpins its potential for treating conditions such as depression [5]. HML is readily absorbed from the gastrointestinal tract, although experimental studies typically employ subcutaneous or intravenous administration. A critical aspect of its pharmacology is the rapid development of tolerance to its tremorgenic effects, the duration of which depends on the route of administration and dosage [6]. To date, harmaline is a controlled or prohibited substance in various countries, except for specific medical or scientific purposes.

The development of effective and safe pharmaceuticals necessitates the mandatory investigation of their pharmacologically important physicochemical properties, such as solubility, distribution in pharmaceutically relevant solvents, and membrane permeability [7,8,9]. Expanding knowledge on the key properties of active pharmaceutical ingredients facilitates the development of prospective targeted delivery systems, which enable dose reduction, thereby lowering toxicity and mitigating adverse side effects. This aspect is particularly important for harmaline, a compound which, alongside its benefits, has serious drawbacks that significantly limit and in some cases completely preclude its use.

A notable scarcity of recent data exists on the physicochemical properties of HML. To date, research has largely focused on specific aspects, such as its spectroscopic interaction with bovine serum albumin [10], the ionization of harmala alkaloids [11], transport across Caco-2 and MDCK cell monolayers [12], and quantitative determination by HPLC [13]. Moreover, several key studies are dated (e.g., 1983, 2000, 2002, 2017, 2019). Crucially, no literature data were found on the solubility of HML in pure aqueous buffers, 1-octanol, *n*-hexane, or isopropyl myristate, or on its distribution coefficients in the corresponding 1-octanol/buffer, *n*-hexane/buffer, and isopropyl myristate/buffer systems. Furthermore, the thermodynamic and kinetic aspects of these processes remain uncharacterized.

**Figure 1:**
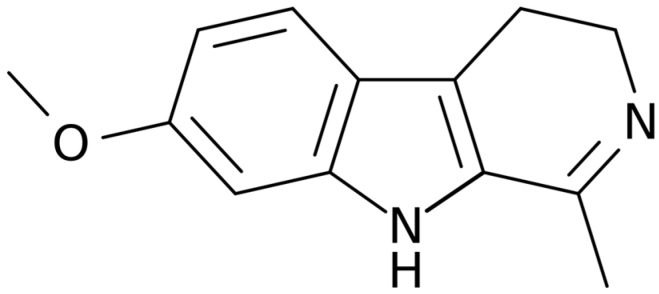
Harmaline structure.

Based on these premises, this work addresses the existing knowledge gap concerning the solubility, distribution, and permeability of HML (Figure 1), with a focus on its thermodynamic and kinetic aspects. The findings are expected to provide a foundation for the compound’s novel therapeutic applications.

## 2. Materials and Methods

### 2.1. Materials

Details of all chemicals used are listed in Table S1, and the buffer preparation protocol can be found in Section S1 of the Supplementary Materials.

### 2.2. Solubility Determination and Modeling

The equilibrium solubility of HML was measured by the shake-flask method over a wide temperature range of 293.15–313.15 K. Briefly, glass vials containing the respective solvent were charged with an excess of the drug. The vials were shaken at a predetermined temperature for 24 h—a duration previously established as sufficient to reach equilibrium between the solid and liquid phases. Following shaking, samples were equilibrated for ~5 h to prevent supersaturation and then centrifuged (10,000 rpm, 10 min; Biofuge pico, Thermo Electron LED GmbH, Osterode am Harz, Germany) at constant temperature. Concentrations were determined spectrophotometrically at 373 nm using a Cary-50 instrument (Varian, Palo Alto, CA, USA; software version 3.00 (339)) based on calibration curves. Data are presented as the mean ± standard deviation (or standard error) of at least three independent replicates. The error bars represent the standard deviation (SD), which ranged from 2% to 4% of the mean.

The temperature dependence of the experimental solubility was modeled via the van’tHoff and modified Apelblat equations [14] useful for quantitatively description of the compound-solvent equilibrium. The correlation equations are provided in Section S2.

### 2.3. Miscibility Evaluation

The miscibility of HML with the solvents was assessed using Hansen solubility parameters (HSP), a method widely applied in pharmaceutical research [15]. The calculations were performed according to the methods of Hoftyzer and Van Krevelen [16,17]. The key equations used are provided in Section S3 (Supplementary Materials).

### 2.4. Determinations of the Distribution Coefficients

The equilibrium distribution coefficients of HML were determined in the OctOH/pH 7.4 and Hex/pH 7.4 systems by the shake-flask method over a wide temperature range of 293.15–313.15 K. The distribution coefficient in the IPM/pH 7.4 system was obtained at 310.15 K. Before the experiment, the aqueous and organic phases were mutually pre-saturated by vigorous shaking for 24 h, equilibrated, and then carefully separated. The stock HML solutions were prepared in OctOH for the OctOH/pH 7.4 system and in pH 7.4 for both the Hex/pH 7.4 and IPM/pH 7.4 systems. The volumes of the organic and aqueous phases were selected according to the HML solubility in each solvent. The glass vials containing the two-phase system were equilibrated at constant temperature for 24 h. After this, the vials were allowed to stand for not less than 4 h, and the phases were carefully separated. The concentration of HML in each phase was measured using a UV-VIS spectrophotometer (Cary-50 Varian, Palo Alto, CA, USA; Software Version 3.00 (339)). Each experiment was carried out in three or more replicates. The standard deviation ranged from 2% to 4% of the mean. The distribution coefficients were calculated by Equation (1):

(1) $$D_{app}^{Org/pH7.4}=\frac{C_{2}^{Org/pH7.4}\cdot V^{pH7.4/Org}}{C_{2}^{pH7.4/Org}\cdot V^{Org/pH7.4}}$$

where $D_{app}^{Org/pH7.4}$ is the distribution coefficient calculated using the compound concentrations in molar units, $C_{2}^{Org/pH7.4}$ and $C_{2}^{pH7.4/Org}$ are the molar concentrations of the drug in the mutually saturated organic and aqueous phases, respectively, $V^{pH7.4/Org}$ and $V^{Org/pH7.4}$ are the volumes of the mutually saturated buffer and organic phases, respectively. The Δlog*D* parameters that characterize the drug’s hydrogen-bonding potential and reflect the impact of specific interactions on partition processes were calculated as follows:

(2) $$\Delta \log D^{Oct/Hex}=\log D_{app}^{Oct/pH7.4}-\log D_{app}^{Hex/pH7.4}$$

(3) $$\Delta \log D^{IPM/Hex}=\log D_{app}^{IPM/pH7.4}-\log D_{app}^{Hex/pH7.4}$$

Distribution coefficients were measured at 1, 2, 3, 4, and 5 h in OctOH/pH 7.4 and IPM/pH 7.4 at 310.15 K to assess the kinetics of HML distribution.

### 2.5. Solubility and Distribution Thermodynamic Parameter Determinations

The calculation of the thermodynamic parameters for the solubility and distribution processes was based on component concentrations expressed in terms of mole fractions. The standard calculation procedure and the respective equations are presented in the Supplementary Materials (Section S4).

### 2.6. Permeability Experiments

Permeability coefficients were measured in a vertical type Franz diffusion cell (PermeGear, Inc., Hellertown, PA, USA) maintained at 37 °C, with an effective diffusion area of 0.785 cm^2^. The volumes of the donor (lower) and the acceptor (upper) compartments were 7 mL and 2 mL, respectively. Three artificial membranes were used in the study: Regenerated cellulose (RC): Standard Grade RC Dialysis Membrane with a molecular weight cut-off (MWCO) of 12–14 kDa and a flat width of 45 mm; PermeaPad barrier (PP): A commercially available barrier (PHABIOC, Fritz-Souchon-Str. 27, 32339 Espelkamp, Germany; www.innome.de), originally proposed by di Cagno et al. [14]; Polydimethylsiloxane-polycarbonate membrane (PDS): A “Carbosil” membrane composed of 55% polydimethylsiloxane and 45% polycarbonate (PENTAMED, Moscow, Russia; https://medpolymer.ru/).

A solution of HML in pH 7.4 (7 mL) was placed into the donor chamber of a Franz cell. The acceptor compartment was filled with pure buffer pH 7.4 (1 mL). Over a period of 5 h, 0.5 mL samples were withdrawn from the acceptor compartment at 30 min intervals and replaced with an equal volume of fresh pH 7.4 buffer. The sample concentrations were determined by UV-Vis spectroscopy (Cary-50, Varian, Palo Alto, CA, USA; Software Version 3.00 (339)) or by HPLC (for experiments involving the polydimethylsiloxane-polycarbonate membrane). The cumulative amount of drug permeated through the membrane was plotted as a function of time. The flux was determined from the slope of the linear portion of each curve. The permeability coefficient was calculated using the following equation:

(4) $$P_{app}=J/C_{0}$$

where *J* is the flux, and *C*_0_ is the concentration of HML in the donor solution. A reverse dialysis setup was employed, where the lower chamber served as the donor compartment and was filled with the drug solution. Sink conditions were maintained in all experiments, meaning the drug concentration in the acceptor compartment never exceeded 10% of its concentration in the donor compartment at any time according the recommendations proposed in [18].

### 2.7. Differential Scanning Calorimetry

Thermal characterization was performed using a differential scanning calorimeter (DSC 4000, PerkinElmer, Shelton, CT, USA) equipped with a refrigerated cooling system. Samples were heated in standard aluminum pans at a rate of 10 K·min^−1^ under a nitrogen atmosphere. The instrument was calibrated using indium and zinc standards. The sample mass was approximately 0.3 mg, weighed with an accuracy of ±0.01 mg. For each sample, a minimum of three replicate experiments were conducted, and the average value was used for the evaluation of the heat of fusion.

### 2.8. HPLC Analysis

Samples from the acceptor compartment after permeation through the PDS membrane were analyzed by HPLC using a Shimadzu Prominence LC-20 AD system(Shimadzu Corporation, Kyoto, Japan) equipped with a PDA detector and a Luna^®^ C-18 column (150 × 4.6 mm i.d., 5 μm particle size, 100 Å pore size). The column temperature was maintained at 40 °C. The mobile phase consisted of acetonitrile and water (containing 0.1% trifluoroacetic acid) in a 20:80 (*v*/*v*) ratio. An isocratic elution was performed at a flow rate of 1.0 mL·min^−1^. The injection volume was 20 μL. The analytes were detected by UV at 373 nm, with a retention time of approximately 4.5 min. Detailed information on the calibration is provided in Section S5 (Supplementary Materials).

## 3. Results and Discussion

### 3.1. Determination of HML Solubility

Literature sources do not provide precise melting point and enthalpy values for HML. In this study we performed DSC analysis and determined the following values: *T_onset_* = 249.0 ± 0.2 °C, *T_peak_* = 254.5 ± 0.2 °C, and ∆*H_m_* = 38.6 ± 0.7 kJ∙mol^−1^. The DSC thermogram is presented in Figure S1a.

To simulate hydrophilic, lipophilic, and non-polar biological regions, the solubility of HML was measured in three common solvents: aqueous buffer pH 7.4, OctOH, and Hex at different temperatures. To simulate gastric and skin barrier environments, solubility was also determined in pH 2.0 and IPM, respectively, at 310.15 K. The solid phases recovered from the solubility experiments were dried and analyzed by DSC (Figure S1b). The analysis showed no evidence of solvate/hydrate formation or polymorphic transformation of HML. The solubility temperature dependences are provided in Table S2. At 310.15 K, the solvents exhibited the following order of HML solubility: OctOH (*S*_2_ = 6.29∙10^−2^ M) > pH 2.0 (*S*_2_ = 1.08∙10^−2^ M) > pH 7.4 (*S*_2_ = 4.99∙10^−3^ M) > IPM (*S*_2_ = 9.44∙10^−4^ M) > Hex (*S*_2_ = 1.30∙10^−4^ M). Compared to other carboline alkaloids, HML exhibits substantially higher solubility at pH 7.4. It is 44-fold more soluble than vincamine, 42-fold more soluble than reserpine, and also surpasses the solubility of other poorly soluble alkaloids such as brovincamine, cipargamine, lurbinectedin, and vinpocetine [19]. Consistent with its weak basic character, HML exhibited 2.2-fold higher solubility in an acidic medium (pH 2.0) than in a neutral one (pH 7.4). This can be explained by its ionization state: at pH 2.0, HML exists predominantly in its protonated (cationic) form, while at pH 7.4, the neutral species prevails [11]. The increased solubility at low pH is attributed to the greater affinity of the cationic species to form hydrogen bonds with polar water molecules [20].

The highest solubility of HML in OctOH (mainly non-polar solvent) can be attributed to several factors. First, this solvent possesses both a polar hydroxyl group capable of forming hydrogen bonds and a long, eight-carbon hydrophobic chain. This amphiphilic structure allows it to interact with both the polar and lipophilic moieties of the HML molecule, aligning with the “like dissolves like” principle.

IPM, an ester of myristic acid and isopropyl alcohol, contains polar ester linkages and a long hydrocarbon chain, which account for its lipophilic character. Its solvation properties are characterized by low dipolarity/polarizability, moderate hydrogen-bond basicity, and negligible hydrogen-bond acidity [21,22]. These specific properties of IPM are responsible for its intermediate position in the solvent series with respect to HML solubility.

Although Hex is a non-polar hydrocarbon solvent (log*P* = 3.5) composed solely of C–C and C–H bonds, which could, in principle, solvate the hydrophobic regions of HML, it exhibited the lowest solubility among all solvents tested. The solubility values in Hex were 484, 38, and 7 times lower than those in OctOH, pH 7.4, and IPM, respectively. This can be attributed to the inert nature of n-hexane, which allows only for non-specific (dispersion, van der Waals) interactions.

To accurately quantify the relationship between experimental solubility and temperature, the mole fraction solubility of HML was fitted to both the van’t Hoff and modified Apelblat equations (Equations (S1) and (S6)). A comparison of the experimental and calculated values, including the relative deviations, is provided in Table S3. Table S4 summarizes the fitted parameters for the modified Apelblat and van’t Hoff equations.

As shown in Tables S3 and S4, both the van’t Hoff and modified Apelblat equations provide a good correlation between the experimental and calculated solubility values for HML. Both models are suitable for correlating the solubility of HML in all solvents studied. However, the van’t Hoff equation showed better correlation performance based on the total average *RD*, *RAD*, and *RMSD* values.

### 3.2. Miscibility of HML with the Studied Solvents

To evaluate drug-solvent miscibility and characterize their interactions, the Hansen approach based on solubility parameters was applied. The calculated group contribution parameters for the studied compound and solvents are presented in Table S5. Table S6 provides the molar volumes (*V*) along with the Hansen solubility parameters: the dispersion component ($\delta_{d}$), the polar component ($\delta_{p}$), and the hydrogen bonding component ($\delta_{h}$). The miscibility of HML with the studied solvents was evaluated based on the parameter of Van Krevelen and Hoftyzer [17] (Equation (S6)). Two compounds are considered miscible if $\Delta \bar{\delta }$ ≤ 5 MPa^0.5^. According to the $\Delta \bar{\delta }$ values, the solvents can be ranked as follows: OctOH (4.6) ˂ IPM (6.7) ˂ Hex (10.3) ˂˂ Buffer (35.9). The solubility results confirm the ranking of the organic solvents, demonstrating a clear correlation between predicted miscibility and experimental solubility. Only OctOH demonstrated substantial miscibility with HML, attributable predominantly to hydrogen bonding (Table S6). The contribution of hydrogen bonding to miscibility was 4.3 times greater for OctOH than for IPM. The $\Delta \bar{\delta }$ value for the aqueous buffer is significantly higher than those of the other solvents, despite its solubility being intermediate between OctOH and IPM. The observed discrepancy is presumably due to the ionized state of HML species. In all likelihood, it is fully charged at pH 2.0 but exists as a mixture of cationic and neutral species at pH 7.4. Consequently, the Hansen approach is not applicable in this case.

### 3.3. Distribution Coefficients of HML in OctOH/pH 7.4, Hex/pH 7.4 and IPM/pH 7.4 Systems

Apparent distribution coefficients of HML were determined in the OctOH/pH 7.4 ($D_{app}^{OctOH/pH7.4}$), Hex/pH 7.4 ($D_{app}^{Hex/pH7.4}$) and IPM/pH 7.4 ($D_{app}^{IPM/pH7.4}$) at various temperatures, with $D_{app}^{IPM/pH7.4}$ measured specifically at 310.15 K. The influence of specific interactions on the partitioning process in the OctOH/pH 7.4 and IPM/pH 7.4 systems was quantified using the ∆log*D* parameter, calculated according to Equations (2) and (3).

The OctOH/pH 7.4 distribution coefficient (a lipophilicity indicator) simulates the compound’s partition between the lipid layer of the biological membranes and the blood flow. The partition coefficient in the Hex/pH 7.4 system serves as a model for penetration through the blood–brain barrier [23]. The IPM/pH 7.4 system, which is commonly used to simulate the stratum corneum/water partition [24], provides relevant information for predicting skin membrane permeation [25]. Notably, IPM is less lipophilic than OctOH, as indicated by their calculated log*P* values (*C*log*P* = 7.25 vs. 2.876, respectively).

The values of $D_{app}$ (log$D_{app}$) are provided in Table 1. Table S7 lists the raw experimental data for the HML molar concentrations in the organic and aqueous phases of the distribution systems.

It is interesting to note that for HML the value of $D_{app}^{Hex/pH7.4}$ = 0.0260 (298.15 K) determined in this study is lower than the values reported elsewhere for the *n*-heptane/aqueous (pH 7.4) system: 0.05 [19] and 0.11 [20], which were measured at an undefined temperature. In addition, according to Kerns and Di’s classification [26], the log$D_{app}^{OctOH/pH7.4}$ values of HML from 0.973 at 293.15 K to 1.345 at 313.15 K, fall within the optimal range for potential bioavailability (log*D* = 1–3). The ∆log*D* value of 2.93 ± 0.03 indicates that HML has greater affinity for lipophilic tissues than for polar biological regions. The distribution coefficient of HML was highly dependent on the nature of the organic phase. At 310.15 K, the values decreased in the order: $D_{app}^{OctOH/pH7.4}$ (19.492) > $D_{app}^{IPM/pH7.4}$ (0.250) > $D_{app}^{Hex/pH7.4}$ (0.022), which is consistent with the solubility trends in the corresponding solvents. The low distribution coefficient for the Hex/Buffer system is due to hexane’s capability for only non-specific interactions with the studied compound. The 2.8-fold higher Δlog*D* value for the OctOH/pH 7.4 system compared to IPM/pH 7.4 suggests that specific interactions, particularly hydrogen bonding, are the primary driving force for HML’s higher distribution in the OctOH system. This conclusion is supported by the Hansen solubility parameter analysis presented in the previous section. In the following section, the driving forces behind this distribution behavior are analyzed from a thermodynamic perspective.

### 3.4. Solubility and Distribution Thermodynamics Evaluation

**Figure 2:**
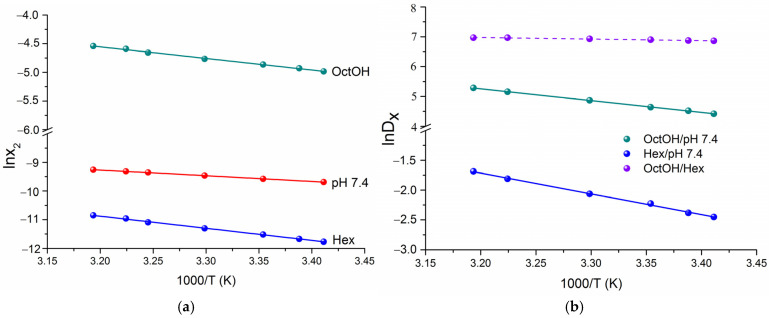
The van’t Hoff plots of experimental solubility (**a**) and distribution coefficient (**b**) of HML in the tested solvents.

To obtain the thermodynamic functions of solubility and distribution, van’t Hoff plots were constructed using mole fraction data on a unitary scale (Figure 2a and Figure 2b, respectively).

**Figure 3:**
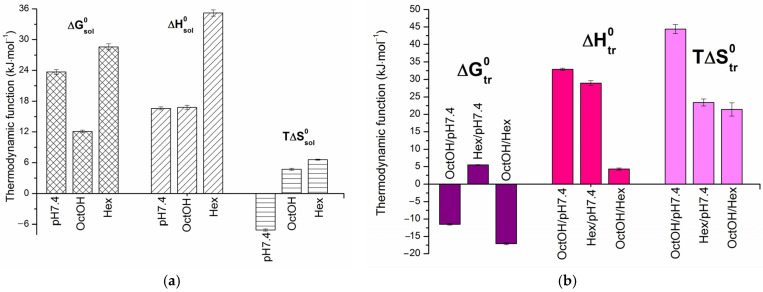
Dissolution (**a**) and distribution (**b**) thermodynamic parameters for the standard temperature of 298.15 K.

The solubility and distribution data followed linear van’t Hoff dependences. The calculated standard thermodynamic parameters for dissolution ($\Delta G_{sol}^{0}$, $\Delta H_{sol}^{0}$ and $T\Delta S_{sol}^{0}$) at 298.15 K are presented in the diagram in Figure 3. The equations for the linear dependencies are provided in Section S6.

As shown in Figure 2a, the solubility of HML increased with temperature. The positive enthalpy of solution values (Figure 3a) indicate an endothermic dissolution process, meaning that the energy gain from solvation is insufficient to overcome the crystal lattice energy. The Gibbs energy of solution for HML confirmed the solubility trend, with OctOH ($\Delta G_{sol}^{0}$ = 12.1 kJ·mol^−1^) followed by pH 7.4 ($\Delta G_{sol}^{0}$ = 23.7 kJ·mol^−1^) and Hex ($\Delta G_{sol}^{0}$ = 28.6 kJ·mol^−1^). For all solvents studied, the dissolution was enthalpy-determined, with the absolute enthalpy contribution exceeding the entropy. The maximum difference was observed in Hex, where enthalpy accounted for 84% of the total free energy change, and entropy for 16%. The positive entropy values in the organic solvents drive the dissolution. In contrast, the negative entropy ($T\Delta S_{sol}^{0}$ ˂ 0) in the buffer solution, likely due to the hydrophobic effect (ordering of water molecules), renders the process entropically unfavorable.

Thermodynamic transfer parameters, obtained from the temperature dependence of distribution coefficients, are typically used to elucidate the mechanisms of partitioning. In this study, we calculate the standard thermodynamic parameters for transfer from pH 7.4 to OctOH ($D_{x}^{OctOH/pH7.4}$), from pH 7.4 to Hex ($D_{x}^{Hex/pH7.4}$) and from Hex to OctOH ($D_{x}^{OctOH/Hex}$) using the van’t Hoff linear regressions shown in Figure 2b. $D_{x}^{Hex/pH7.4}$ represents the mole fraction-based analog of ∆log*D* (molar scale), derived from HML concentrations. The values of $D_{x}$ and thermodynamic functions at 298.15 K, along with the corresponding equations, are provided in Table S8. Figure 3b presents a comparison of the thermodynamic transfer parameters across the studied systems. The partitioning of HML is spontaneous from pH 7.4 to OctOH ($\Delta G_{tr}^{0}$ = −11.5 kJ·mol^−1^) and from Hex to OctOH ($\Delta G_{tr}^{0}$ = −17.1 kJ·mol^−1^), as indicated by the negative Gibbs free energy values. Conversely, the transfer from pH 7.4 to Hex ($\Delta G_{tr}^{0}$ = +5.5 kJ·mol^−1^) is unfavorable due to its positive $\Delta G_{tr}^{0}$ value. The enthalpy and entropy contributions to $\Delta G_{tr}^{0}$ exhibit distinct patterns across the different systems. The transfers pH 7.4→OctOH and Hex→OctOH are entropy-determined and driven with entropy contributions of 57.4% and 83.3%, respectively. This fact clearly demonstrated the hydrophobic effect and its role in enhancing the overall disorder of the system. The larger value of $T\Delta S_{tr}^{0}$ for the OctOH/pH 7.4 system is likely due to molecular aggregates and substructures that disrupt the water-saturated OctOH microstructure, thereby increasing entropy. As is evident from Figure 3b, the enthalpy (55.3%) and entropy (44.7%) contributions in the Hex/pH 7.4 system are comparable, with enthalpy being slightly more dominant.

### 3.5. Membrane Permeability of HML

To complete the physicochemical profile of HML, we measured the permeability through different types of artificial membranes. PDS membrane effectively mimics drug permeability across the human epidermis, making it a widely used in vitro model for human skin [27]. The PP barrier, which consists of a lipid layer sandwiched between two support layers, is a relatively new tool for assessing permeability. Upon contact with an aqueous solution, the lipid layer forms tightly packed vesicles that structurally resemble a biological membrane [28]. It has been proposed as a model for simulating absorption across the buccal or intestinal epithelium [18]. As shown in Section 3.3, the partition coefficients obtained from OctOH/pH 7.4 and IPM/pH 7.4 systems serve as models for intestinal membrane and stratum corneum permeability, respectively. Establishing a correlation between these partition coefficients and permeability parameters is useful for developing predictive models. Such models can forecast the properties of newly synthesized compounds, particularly within series of structural analogs, and aid in the design of effective drug formulations.

**Figure 4:**
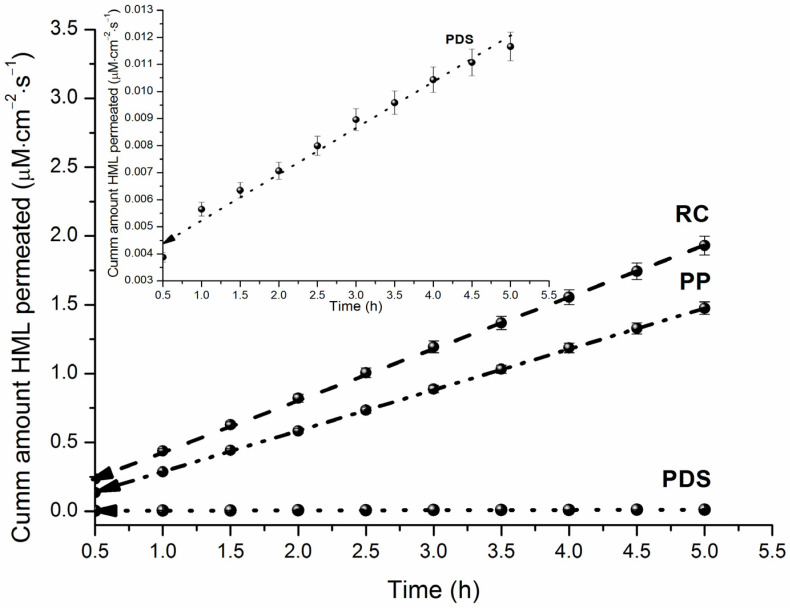
HML cumulative amount permeated across RC, PDS and PP at 310.15 K.

The hydrophilic RC membrane is considered a standard barrier due to its simplicity and low cost. This membrane is characterized by a shorter lag time, higher flux values, and good permeability for many substances, making it suitable for studying poorly soluble compounds. It is often used in transport studies to compare different drug formulations [29]. Harmaline has low aqueous solubility, which necessitates its formulation using pharmaceutical excipients (e.g., cyclodextrins, biopolymers, micelles). For preliminary permeability screening in the presence of such solubilizing additives, a cellulose membrane can be employed. The apparent permeability coefficients (*P_app_*), donor solution concentrations (*C*_0_), and steady-state fluxes (*J*) are provided in Table 2. The cumulative amount of permeated HML is shown as a function of time in Figure 4.

The membranes can be ranked by permeability of HML as RC > PP > PDS, with the permeability coefficients for PP and PDS being 1.3-fold and 299-fold lower, respectively, than that for RC. Following the classification of Di Cagno et al. [18], HML is a high permeated substance (*P_app_* > 2.04∙10^−5^ cm∙s^−1^). The low permeability observed with the PDS membrane can be attributed to its complex heterophase and heteropolar structure [25]. We obtained literature values for HML permeability across Caco-2 monolayers and MDCK cells [12]: *P_Caco-2_* = (2.557–3.098)∙10^−5^ cm∙s^−1^ and *P_MDCK_* = (1.581–2.453)∙10^−5^ cm∙s^−1^, respectively. From these values, it can be concluded that the Caco-2 model permeability is consistent with the PermeaPad results (maximum difference is 1.4-fold), while the MDCK permeability coefficient is up to 2.3-fold lower.

### 3.6. Distribution/Permeability Interrelations

As previously discussed, the PP barrier models intestinal permeability, while the PDS membrane mimics skin penetration. Given that the OctOH/pH 7.4 system represents transport from the intestinal lumen to the blood, and the IPM/pH 7.4 system simulates partitioning into the stratum corneum [30], we compared the distribution and permeation trends across these systems. Based on data from Table 1 (distribution coefficients) and Table 2 (permeability coefficients), we observed that the distribution coefficient decreased by 78-fold from the OctOH/pH 7.4 to the IPM/pH 7.4 system, and the permeability coefficient decreased by 299-fold from the PP to the PDS barrier. While both parameters exhibit similar trends, the reduction in permeability is significantly greater than that in distribution.

**Figure 5:**
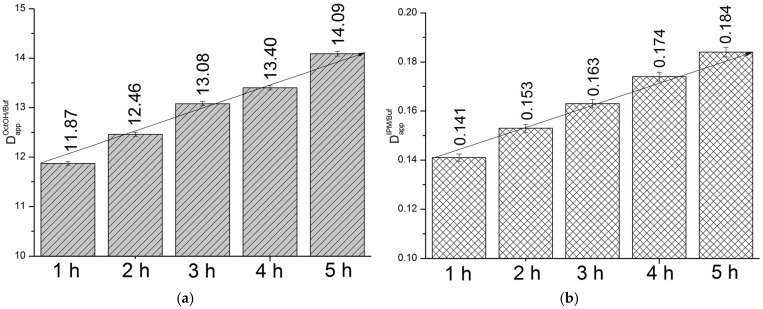
Distribution profiles for OctOH/pH 7.4 (**a**) and IPM/pH 7.4 (**b**) systems, 310.15 K.

We determined the distribution kinetics in the OctOH/pH 7.4 and IPM/pH 7.4 systems at the same time points as the permeation studies (1, 2, 3, 4, and 5 h). Figure 5a,b present the time-dependent distribution profiles for the two systems.

As shown in Figure 5, distribution increased from 1 to 5 h by 1.2-fold in the OctOH/pH 7.4 system and 1.3-fold in the IPM/pH 7.4 system. Considering the values of the thermodynamic distribution coefficients at 310.15 K ($D_{app}^{OctOH/pH7.4}$ = 19.49 and $D_{app}^{IPM/pH7.4}$ = 0.25), equilibrium is not reached in either system within 5 h. Notably, both systems exhibited a comparable difference between the saturation and 5 h distribution coefficients, with fold-changes of 1.38 and 1.36, respectively. The temporal evolution of the distribution coefficients over 5 h followed linear trends, described by the following equations:

(5) $$D_{app}^{OctOH/pH7.4}=11.37(\pm 0.10)\;+\;1.49\cdot 10^{-4}(\pm 8.00\cdot 10^{-6})\cdot t(s),\;r=0.9957,\;n=5$$

(6) $$D_{app}^{IPM/pH7.4}=0.13(\pm 4.72\cdot 10^{-4})\;+\;2.94\cdot 10^{-6}(\pm 3.95\cdot 10^{-8})\cdot t(s),\;r=0.9997,\;n=5$$

**Figure 6:**
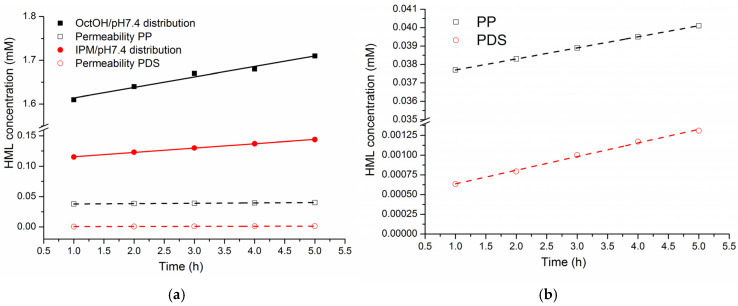
Kinetic dependences of HML concentration in the organic phases of distribution systems and in the acceptor cell during permeation: (**a**) distribution and permeability, (**b**) permeability on a large scale.

The kinetic profiles of HML concentration in the OctOH and IPM phases, measured at hourly intervals over 5 h, were analyzed and compared with its concurrent accumulation in the acceptor compartment following permeation through the PP and PDS membranes. The corresponding concentration data are provided in Table S9, and the kinetic profiles are illustrated in Figure 6.

As shown in Figure 6, all concentration profiles followed linear regression trends, with a steeper slope for the distribution processes than for permeation. Compared to the IPM/PDS system, the OctOH/PP system showed a more pronounced increase in concentration in both the organic phase and the acceptor compartment. These results suggest that the OctOH/pH 7.4/PP and IPM/pH 7.4/PDS systems serve as suitable models for the in vitro evaluation of HML transport properties.

## 4. Conclusions

This work presents new data for a beta-carboline harmala alkaloid HML, including its solubility and solution thermodynamics in aqueous buffers (pH 2.0 and pH 7.4), OctOH, Hex, and IPM; distribution and transfer thermodynamics in OctOH/pH 7.4, Hex/pH 7.4 and IPM/pH 7.4 systems; and permeability across RC, PDS and PP membranes. At 310.15 K, the molar solubility of HML in the solvents decreased in the following order: OctOH (*S*_2_ = 6.29∙10^−2^ M) > pH 2.0 (*S*_2_ = 1.08∙10^−2^ M) > pH 7.4 (*S*_2_ = 4.99∙10^−3^ M) > IPM (*S*_2_ = 9.44∙10^−4^ M) > Hex (*S*_2_ = 1.30∙10^−4^ M). The compound’s solubility in organic solvents conformed to the calculated Hansen solubility parameters ($\Delta \bar{\delta }$). The value of the OctOH/pH 7.4 distribution coefficient for HML meets the optimal criteria for potential bioavailability. Based on the distribution data, HML exhibits preferential partitioning into lipophilic tissues over polar biological matrices.

Regarding its permeability across the PP, HML was classified as highly permeable, with a *P_app_* (PP) = 3.71∙10^−5^ cm∙s^−1^. This value is very close to its permeability across Caco-2 monolayers reported in the literature. The kinetic behavior of distribution and permeability was characterized and discussed for a period of up to 5 h. The analogous trends in the concentration profiles of HML in the OctOH/pH 7.4—PP and IPM/pH 7.4—PDS systems validate their use as appropriate models for in vitro evaluation of HML transport properties.

This work advances the understanding of harmaline’s pharmacologically relevant properties, thereby facilitating solutions to persistent challenges in its pharmacological application.

## Figures and Tables

**Table 1:** Distribution coefficients of HML and ∆logD parameter from 293.15 K to 313.15 K.

*T*(K)	$D_{app}^{OctOH/pH7.4}$ (log*D*)	$D_{\mathrm{app}}^{\mathrm{Hex}/\mathrm{pH}7.4}$·10^2^(log*D*)	∆log*D*	$D_{app}^{IPM/pH7.4}$ (log*D*)	∆log*D*
293.15	9.399 ± 0.101 (0.973)	1.178 ± 0.024 (−1.929)	2.90	-	-
298.15	10.376 ± 0.208 (1.016)	1.260 ± 0.0.25 (−1.899)	2.92	-	-
303.15	11.653 ± 0.233 (1.066)	1.470 ± 0.037 (−1.833)	2.90	-	-
308.15	14.632 ± 0.293 (1.165)	1.718 ± 0.035 (−1.765)	2.93	-	-
310.15	19.492 ± 0.389 (1.290)	2.197 ± 0.044 (−1.658)	2.95	0.250 ± 0.010 (−0.602)	1.056
313.15	22.138 ± 0.443 (1.345)	2.483 ± 0.050 (−1.605)	2.95	-	-

The standard uncertainties are *u*(*m*) = 0.01 mg, *u*(*T*) = 0.15 K and *u*(*p*) = 3 kPa. The combined expanded uncertainties are *U_c_*(*logD*_C_) = 0.07*logD*_C_.

**Table 2:** Donor solution concentrations (*C*_0_), steady state flux (*J*), and permeability coefficients (*P_app_*) of HML in pH 7.4 at 310.15 K.

Membrane	*C*_0_ (M)	*J* (µmol∙cm^−2^∙s^−1^)	*P_app_* (cm∙s^−1^)	log*P_app_*
RC	2.11 × 10^−3^	1.04 × 10^−4^	(4.93 ± 0.17) × 10^−5^	−4.31
PDS	2.62 × 10^−3^	4.31 × 10^−7^	(1.65 ± 0.07) × 10^−7^	−6.78
PP	2.23 × 10^−3^	8.28 × 10^−5^	(3.71 ± 0.11) × 10^−5^	−4.43

## Data Availability

The results obtained for all experiments performed are shown in the manuscript and Supplementary Materials; all samples are available from the author upon reasonable request.

## Supplementary Materials

The following supporting information can be downloaded at: https://www.mdpi.com/article/10.3390/pharmaceutics18010035/s1, can be downloaded at: https://www.mdpi.com/, Table S1: Chemicals used in the present study; Section S1: Buffer preparation procedure; Section S2: Modeling using the van’t Hoff and modified Apelblat equations; Section S3: Determination of Hansen solubility parameters; Section S4: Thermodynamic parameters determinations; Section S5: HPLC calibration; Figure S1: The DSC thermogram of raw HML (a) and solid residuals after dissolution in solvents (b); Table S2: Temperature dependence of solubility, *S*_2_ (M) of HML in a buffer at pH 7.4, OctOH, Hex; Table S3: Experimental ($x_{2}^{\exp}$) and correlated ($x_{2}^{cal}$) mole fractions solubility of HML in the selected solvents at different temperatures; Table S4: The parameters of the van’t Hoff and modified Apelblat equations calculated for HML solubility in the investigated solvents; Table S5: The group contribution parameters of HML; Table S6: Hansen solubility parameters of HML, investigated solvents and evaluative parameter $\Delta \bar{\delta }$; Table S7: Molar concentration (*C*_2_) of HML and the respective volumes of the organic and aqueous phases; Section S6: Linear regression of solubility vs. temperature; Table S8: Distribution coefficients of HML (mole fraction scale) and the standard thermodynamic functions at 298.15 K for Buffer→OctOH, Buffer→Hex and Hex→OctOH transfer; Table S9: HML concentration in the organic phases of distribution systems and the acceptor cell during permeation. References [31,32,33] are cited in the Supplementary Materials.
