# Supplementary material for: Bridging the Knowledge Gap in Harmaline’s Pharmacological Properties: A Focus on Thermodynamics and Kinetics
Source: Pharmaceutics. 2025 Dec 26;18(1):35. doi: 10.3390/pharmaceutics18010035 (PMC12844998; doi:10.3390/pharmaceutics18010035)
Supplement: Supplementary file 1 [file pharmaceutics-18-00035-s001.zip › pharmaceutics-4048843-supplementary.pdf]

Supporting Information

Bridging the knowledge gap in harmaline's pharmacological properties: A focus on thermodynamics and kinetics

Tatyana V. Volkova*, Olga R. Simonova, German L. Perlovich

G.A. Krestov Institute of Solution Chemistry RAS, 153045 Ivanovo, Russian Federation
**Corresponding author: 1 Akademicheskaya str., 153045 Ivanovo, Russian Federation,*
E-mail: vtv@isc-ras.ru

Table S1. Chemicals used in the present study.

Compound	CAS No.	Source	Mass Fraction Purity
Harmaline (HML)	304-21-2	Sigma-Aldrich (St. Louis, USA, Missouri)	≥95%
1-Octanol (OctOH)	111-87-5	Sigma-Aldrich (St. Louis, USA, Missouri)	≥99%
n-Hexane (Hex)	110-54-3	Sigma-Aldrich (St. Louis, USA, Missouri)	≥97%
Isopropyl myristate (IPM)	110-27-0	BLDpharm	98%
Potassium dihydrogen phosphate	7778-77-0	Merk (Darmstadt, Germany)	≥99%
Disodium hydrogen phosphate dodecahydrate	10039-32-4	Merk (Darmstadt, Germany)	≥99%
Potassium chloride	7447-40-7	Merk (Darmstadt, Germany)	≥99%
Acetic acid	64-19-7	Merk (Darmstadt, Germany)	≥99%
Sodium hydroxide	1310-73-2	Merk (Darmstadt, Germany)	≥97%
Hydrochloric acid 0.1 mol·dm^{-3} fixanal	7647-01-0	Sigma-Aldrich (St. Louis, USA, Missouri)	-

Section S1. Buffer preparation procedure

Phosphate buffer pH 7.4 was prepared using KH_2PO_4 (9.1 g in 1 L) and $Na_2HPO_4 \cdot 12H_2O$ (23.6 g in 1 L) salts. Acetate buffer at pH 5.0 was prepared at room temperature by dissolving 4.040 g of NaOH (pellets), 8.650 g of CH_3COOH, and 11.87 g NaCl in 1 L of purified deionized water. Buffer solution pH 2.0 was made of 6.57 g of KCl dissolved in water with the addition V=119.0 mL of 0.1 mol·L^{-1} hydrochloric acid. The volume of the result solution was adjusted to 1 L with water.

Double distilled water with an electrical conductivity of 2.1 μS·cm^{-1} (PWT H198308, HANNA® instruments) was used for the preparation of the solutions.

A FG2-Kit pH meter (Mettler Toledo, Switzerland) standardized with pH 4.00 and 7.00 solutions was used to measure and controlling the buffer solutions pH.

Section S2. Modeling using the van't Hoff and modified Apelblat equations

The mole fraction solubility of HML (x_2) was determined for each solvent and subsequently modeled using both the van't Hoff and modified Apelblat equations:

$$\ln x_2 = A + \frac{B}{(T/K)} + C\ln(T/K) \quad \text{(S1)}$$

where T is the absolute temperature, A, B and C are the empirical model parameters. Parameters A and B represent the variation in solution behavior due to the non-ideality of the solute, whereas parameter C reflects the dependence of the enthalpy of fusion on temperature [1].

The validity and accuracy of the applied models were assessed by calculating the relative average deviation (RAD) and root-mean-square deviation ($RMSD$):

$$RAD = \frac{1}{N}\sum_{i=1}^{N}\left|\frac{x_2^{\exp} - x_2^{cal}}{x_2^{\exp}}\right| \quad \text{(S2)}$$

$$RMSD = \left|\frac{1}{N}\sum_{i=1}^{N}(x_2^{\exp} - x_2^{cal})^2\right|^{1/2} \quad \text{(S3)}$$

where N is the number of the experimental points, $x_2^{\exp}$ and x_2^{cal} - the experimental and calculated mole fraction solubility of HML, respectively.

Section S3. Determination of Hansen solubility parameters

The key equations used for the miscibility evaluation are as follows:

$$\delta_d = \frac{\Sigma F_{di}}{V},\ \delta_p = \frac{\sqrt{\Sigma F_{pi}^2}}{V},\ \delta_h = \sqrt{\frac{\Sigma E_{hi}}{V}} \quad \text{(S4)}$$

where δ_d, δ_p, and δ_h are the contributions from the dispersion forces, polar forces, and hydrogen bond energy, respectively; F_{di}, F_{pi}, and F_{hi} are the dispersion, polar and hydrogen bond energy providing the molar attraction constants, V is the molar volume. The contributions δ_d, δ_p, and δ_h were calculated in the following way:

$$\delta_d = \sum F_{di} / \sum V_i,\ \delta_p = (\sum F_{pi}{}^2)^{1/2} / \sum V_i,\ \delta_h = (\sum F_{hi} / \sum V_i)^{1/2} \quad \text{(S5)}$$

The $\Delta\overline{\delta}$ factor reflecting the miscibility was determined by the equation:

$$\Delta\overline{\delta} = \left[(\delta_{d2} - \delta_{d1})^2 + (\delta_{p2} - \delta_{p1})^2 + (\delta_{h2} - \delta_{h1})^2\right]^{0.5} \quad \text{(S6)}$$

Section S4. Thermodynamic parameters determinations

The apparent thermodynamic functions for the dissolution of HML in the solvents were estimated using the van't Hoff equation:

$$\ln x_2 = -\frac{\Delta H^o_{sol}}{RT} + \frac{\Delta S^0_{sol}}{R} \qquad \text{(S7)}$$

where x_2 is the HML mole fraction concentration, ΔH^0_{sol} and ΔS^0_{sol} are the apparent standard dissolution enthalpy and entropy, respectively, referring to the standard temperature of 298.15 K; R is the universal gas constant. The apparent Gibbs energy of the solubility processes $\Delta_{dis}G^0$ at 298.15 K was calculated by the following equation:

$$\Delta G^0_{sol} = -RT(\ln x_2^{298.15K}) \qquad \text{(S8)}$$

Distribution thermodynamics was quantitatively evaluated using mole fraction distribution coefficients ($D_x^{Org/buf}$):

$$D_x^{Org/buf} = \frac{x_2^{Org/buf}}{x_2^{buf/Org}} \qquad \text{(S9)}$$

where $x_2^{Org/buf}$ and $x_2^{buf/Org}$ are the molar fractions of the compound in the organic and aqueous phases, respectively. The standard Gibbs free energy of transfer from the buffer to the organic phase (ΔG^0_{tr}) was calculated as follows:

$$\Delta G^0_{tr} = -RT(\ln D_x^{Org/buf}) \qquad \text{(S10)}$$

The standard enthalpy change (ΔH^0_{tr}) and the standard entropy change (ΔS^0_{tr}) upon the transferring process were calculated using the integral form of the van't Hoff's equation:

$$\ln D_x^{Org/buf} = -\frac{\Delta H^0_{tr}}{RT} + \frac{\Delta S^0_{tr}}{R} \qquad \text{(S11)}$$

The slope of $\ln D_x^{Org/buf}$ on $1/T$ dependence reflects ($-\Delta H^0_{tr}/RT$), and the intercept - $\Delta S^0_{tr}/R$.

Section S5. HPLC calibration

Three stock solutions ($1.19\cdot10^{-4}$ M, $1.27\cdot10^{-4}$ M and $1.22\cdot10^{-4}$ M) were prepared in eluent and subsequently diluted to obtain a series of solutions with concentrations ranging from $6.11\cdot10^{-7}$ M and $1.22\cdot10^{-5}$ M. All examined solutions fell within this concentration range. The measurements were performed in triplicate (or more), and the average was calculated. The linear plot with R^2=0.9953 was obtained and described by the following regression equation:

$$y=2.737(\pm0.847)\cdot10^{-7} + 3.809(\pm0.068)\cdot10^{-11}\cdot x \qquad \text{(S12)}$$

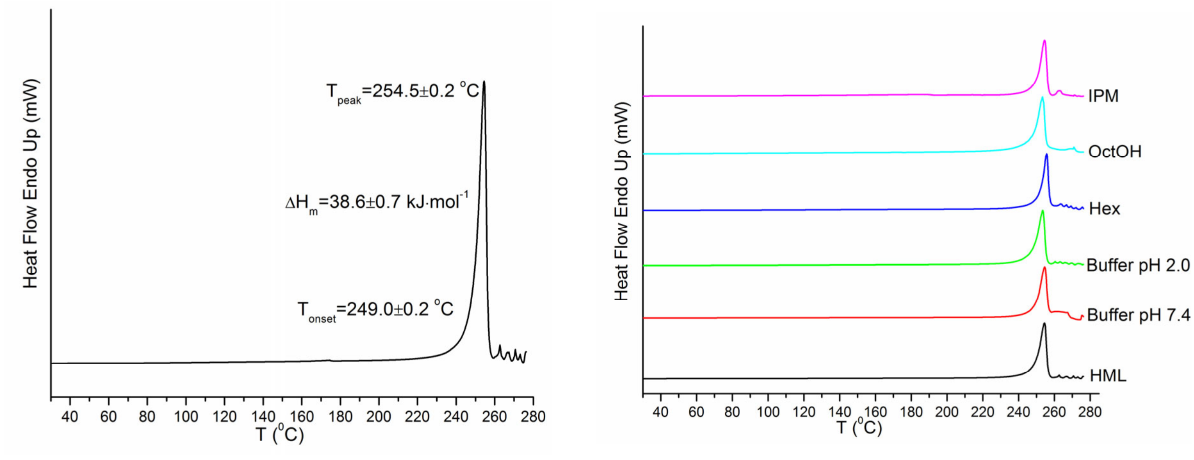

(a) (b)

Figure S1. The DSC thermogram of raw HML (a) and solid residuals after dissolution in solvents (b).

Table S2. Temperature dependence of solubility, S_2 (M) of HML in pH 7.4, OctOH, Hex.

T (K)	pH 7.4	OctOH	Hex	IPM	pH 2.0
	$S_2 \cdot 10^3$	$S_2 \cdot 10^2$	$S_2 \cdot 10^5$	$S_2 \cdot 10^4$	$S_2 \cdot 10^2$
293.15	3.45±0.07	4.32±0.08	5.92±0.13	-	-
295.15	-	4.55±0.09	6.50±0.11	-	-
298.15	3.87±0.06	4.84±0.11	7.53±0.12	-	-
303.15	4.32±0.09	5.32±0.11	9.32±0.17	-	-
308.15	4.82±0.05	5.91±0.13	11.39±0.07	-	-
310.15	4.99±0.05	6.29±0.17	13.00±0.32	9.44±0.20	1.08±0.03
313.15	5.29±0.11	6.58±0.05	14.49±0.41	-	-

Table S3. Experimental ($x_2^{\exp}$) and correlated (x_2^{cal}) mole fractions solubility of HML in the selected solvents at different temperatures.

T (K)	$x_2^{\exp}$	Modified Apelblat equation		van't Hoff equation	
		x_2^{cal}	RD^a	x_2^{cal}	RD^a
Buffer pH 7.4					
293.15	$6.19\cdot10^{-5}$	$6.20\cdot10^{-5}$	-0.0017	$6.20\cdot10^{-5}$	-0.0024
298.15	$6.93\cdot10^{-5}$	$6.97\cdot10^{-5}$	-0.0049	$6.95\cdot10^{-5}$	-0.0031
303.15	$7.75\cdot10^{-5}$	$7.79\cdot10^{-5}$	-0.0048	$7.77\cdot10^{-5}$	-0.0022
308.15	$8.68\cdot10^{-5}$	$8.66\cdot10^{-5}$	0.0018	$8.64\cdot10^{-5}$	0.0036
310.15	$8.99\cdot10^{-5}$	$9.02\cdot10^{-5}$	-0.0035	$9.01\cdot10^{-5}$	-0.0025
313.15	$9.54\cdot10^{-5}$	$9.58\cdot10^{-5}$	-0.0049	$9.59\cdot10^{-5}$	-0.0055
OctOH					
293.15	$6.85\cdot10^{-3}$	$7.01\cdot10^{-3}$	-0.0234	$6.84\cdot10^{-3}$	0.00135
295.15	$7.30\cdot10^{-3}$	$7.32\cdot10^{-3}$	-0.0133	$7.17\cdot10^{-3}$	0.0086
298.15	$7.71\cdot10^{-3}$	$7.83\cdot10^{-3}$	-0.0151	$7.68\cdot10^{-3}$	0.0041
303.15	$8.52\cdot10^{-3}$	$8.74\cdot10^{-3}$	-0.0255	$8.59\cdot10^{-3}$	-0.0077
308.15	$9.50\cdot10^{-3}$	$9.76\cdot10^{-3}$	-0.0269	$9.57\cdot10^{-3}$	-0.0068
310.15	$1.01\cdot10^{-2}$	$1.02\cdot10^{-2}$	-0.0054	$9.98\cdot10^{-3}$	0.0160
313.15	$1.06\cdot10^{-2}$	$1.09\cdot10^{-2}$	-0.0236	$1.06\cdot10^{-2}$	0.0020
Hex					
293.15	$7.74\cdot10^{-6}$	$8.04\cdot10^{-6}$	-0.0385	$7.73\cdot10^{-6}$	0.0019
295.15	$8.51\cdot10^{-6}$	$8.83\cdot10^{-6}$	-0.0364	$8.52\cdot10^{-6}$	-0.0004
298.15	$9.91\cdot10^{-6}$	$1.02\cdot10^{-5}$	-0.0238	$9.84\cdot10^{-6}$	0.0073
303.15	$1.24\cdot10^{-5}$	$1.28\cdot10^{-5}$	-0.0363	$1.24\cdot10^{-5}$	-0.0073
308.15	$1.52\cdot10^{-5}$	$1.61\cdot10^{-5}$	-0.0591	$1.56\cdot10^{-5}$	-0.0259
310.15	$1.74\cdot10^{-5}$	$1.76\cdot10^{-5}$	-0.0139	$1.70\cdot10^{-5}$	0.0207
313.15	$1.95\cdot10^{-5}$	$2.02\cdot10^{-5}$	-0.0377	$1.94\cdot10^{-5}$	0.0037

[a]RD is the relative deviation: $RD = (x_2^{\exp} - x_2^{cal}) / x_2^{\exp}$

Standard uncertainities: $u(T)$ = 0.15 K and $u(p)$ = 3 kPa.
Relative standard uncertainties for solubility: $u_r(X_2)$ = 0.045

Table S4. The parameters of the van't Hoff and modified Apelblat equations calculated for HML solubility in the investigated solvents.

Solvents	*A*	*B*	*C*	*RMSD*	*RAD*
van't Hoff equation					
pH 7.4	-2.86	-2001.76	-	$7.15\cdot10^{-7}$	0.0191
OctOH	1.90	-2018.28	-	$2.00\cdot10^{-4}$	0.0448
Hex	2.66	-4230.40	-	$5.47\cdot10^{-7}$	0.0640
Modified Apelblat equation					
pH 7.4	37.29	-3813.48	-5.98	$7.77\cdot10^{-7}$	0.0216
OctOH	-88.30	2049.89	13.44	$4.16\cdot10^{-4}$	0.1119
Hex	-143.18	2347.42	21.73	$1.18\cdot10^{-6}$	0.2134

Table S5. The group contribution parameters of HML.

Functional group[1,2]	n	F_{di}, $(J/m^3)^{1/2}\cdot mol^{-1}$	F_{pi}, $(J/m^3)^{1/2}\cdot mol^{-1}$	E_{hi}, J/mol
Phenylene	1	1270.0	110.0	0
$-CH_2-$	2	270.0	0	0
$- CH_3$	2	420.0	0	0
=C<	3	70.0	0	0
-O-	1	100.0	400.0	3000.0
-N=	1	20.0	800.0	5000.0
HN<	1	160.0	210.0	3100.0

[1,2] - taken from the literature [2,3]

Table S6. Hansen solubility parameters of HML, investigated solvents and evaluative parameter $\Delta\bar{\delta}$.

Sample	V($cm^3\cdot mol^{-1}$)	$^a\delta_d$ ($MPa^{0.5}$)	$^b\delta_p$ ($MPa^{0.5}$)	$^c\delta_h$ ($MPa^{0.5}$)	$^d\ \Delta\bar{\delta}$
HRM	170.5	18.4	5.4	8.1	-
Buffers	18.0	15.5	16.0	42.3	35.9
OctOH	157.7	17.0	3.3	11.9	4.6
Hex	131.6	14.9	0.0	0.0	10.3
IPM	313.0	15.9	2.1	2.8	6.7

$^a\delta_d = \sum F_{di} / \sum V_i$; $^b\delta_p = (\sum F_{pi}{}^2)^{1/2} / \sum V_i$; $^c\delta_h = (\sum F_{hi}\ / \sum V_i)^{1/2}$; $^d\delta_t = (\delta_d^2 + \delta_p^2 + \delta_h^2)^{1/2}$;

$^d\Delta\bar{\delta} = \left[(\delta_{d2} - \delta_{d1})^2 + (\delta_{p2} - \delta_{p1})^2 + (\delta_{h2} - \delta_{h1})^2\right]^{0.5}$

Table S7. Molar concentration (C_2) of HML and the respective volumes of the organic and aqueous phases.

T (K)	$C_2^{Org/Buf}$ (M)	$C_2^{Buf/Org}$ (M)	C_2^0 (M)	V_{Org}/V_{buf}
OctOH/buffer system				
293.15	$7.76\cdot 10^{-4}$	$5.78\cdot 10^{-4}$	$1.35\cdot 10^{-3}$	2/14
298.15	$8.08\cdot 10^{-4}$	$5.45\cdot 10^{-4}$	$1.35\cdot 10^{-3}$	
303.15	$1.00\cdot 10^{-3}$	$6.03\cdot 10^{-4}$	$1.61\cdot 10^{-3}$	
308.15	$1.09\cdot 10^{-3}$	$5.20\cdot 10^{-4}$	$1.61\cdot 10^{-3}$	
310.15	$9.96\cdot 10^{4}$	$3.58\cdot 10^{-4}$	$1.35\cdot 10^{-3}$	
313.15	$1.03\cdot 10^{-3}$	$3.25\cdot 10^{-4}$	$1.35\cdot 10^{-3}$	
Hex/pH 7.4 system				
293.15	$4.0828\cdot 10^{-6}$	$1.0402\cdot 10^{-3}$	$1.0443\cdot 10^{-3}$	3/9
298.15	$4.3703\cdot 10^{-6}$	$1.0399\cdot 10^{-3}$	$1.0443\cdot 10^{-3}$	3/9
303.15	$1.5526\cdot 10^{-5}$	$1.0562\cdot 10^{-3}$	$1.0717\cdot 10^{-3}$	4/4
308.15	$1.0351\cdot 10^{-5}$	$1.2049\cdot 10^{-3}$	$1.2153\cdot 10^{-3}$	3/6
310.15	$2.4152\cdot 10^{-5}$	$1.0995\cdot 10^{-3}$	$1.1236\cdot 10^{-3}$	4/4
313.15	$2.5302\cdot 10^{-5}$	$1.0190\cdot 10^{-3}$	$1.0443\cdot 10^{-3}$	4/4

IPM/pH 7.4 system				
310.15	$1.85\cdot10^{-4}$	$7.42\cdot10^{-4}$	$9.28\cdot10^{-4}$	3/3

Section S5. Linear regression of solubility vs. temperature

Buffer pH 7.4

$\ln x_2$=-(2.9±0.1)-(2002±18)/T; r=0.9998; σ=$4.47\cdot10^{-5}$; n=6;

OctOH

$\ln x_2$=(1.9±0.1)-(2018±44)/T; r=0.9988; σ=$4.23\cdot10^{-4}$; n=7;

Hex

$\ln x_2$=(2.7±0.2)-(4230±75)/T; r=0.9992; σ=$1.22\cdot10^{-3}$; n=7;

Table S8. Distribution coefficients of HML (mole fraction scale) and the standard thermodynamic functions at 298.15 K for pH 7.4→OctOH, pH 7.4→Hex and Hex→OctOH transfer.

Transfer system	D_x	ΔG^0_{tr} (kJ·mol^{-1})	ΔH^0_{tr} (kJ·mol^{-1})	$T\Delta S^0_{tr}$ (kJ·mol^{-1})	$\Delta\zeta^0_{Htr}$ [4] (%)	$\Delta\zeta^0_{TStr}$ [5] (%)
pH 7.4→ OctOH[1]	103.10	-11.5±0.2	32.9±0.3	44.4±1.3	42.6	57.4
pH 7.4→ Hex [2]	$1.08\cdot10^{-1}$	5.5±0.1	28.9±0.7	23.4±1.0	55.3	44.7
Hex → OctOH[3]	993.14	-17.1±0.3	4.3±0.3	21.4±1.9	16.7	83.3

[1] $\ln D_x^{OctOH/Buf}$=(17.9±0.1)-(3963±34)/T; r=0.9997; σ=$1.79\cdot10^{-4}$; n=6;

[2] $\ln D_x^{Hex/Buf}$=(9.6±0.3)-(3481±81)/T; r=0.9989; σ=$1.03\cdot10^{-3}$; n=6;

[3] $\ln D_x^{OctOH/Hex}$=(8.6±0.1)-(522±38)/T; r=0.9867; σ=$1.79\cdot10^{-4}$; n=6;

[4] $\Delta\zeta^0_{Htr} = |\Delta H^0_{tr}|/(|\Delta H^0_{tr}|+|T\Delta S^0_{tr}|)\times100\%$;

[5] $\Delta\zeta^0_{TStr} = |T\Delta S^0_{tr}|/(|\Delta H^0_{tr}|+|T\Delta S^0_{tr}|)\times100\%$;

Table S9. HML concentration in the organic phases of distribution systems and the acceptor cell during permeation.

Time (h)	C_{ph} [1] (OctOH) (M)	C_{ph} [1] (IPM) (M)	C_{ac} [2] (PP) (M)	C_{ac} [2] (PDS) (M)
1.0	$1.61\cdot10^{-3}$	$1.15\cdot10^{-4}$	$3.77\cdot10^{-5}$	$6.34\cdot10^{-7}$
2.0	$1.64\cdot10^{-3}$	$1.23\cdot10^{-4}$	$3.83\cdot10^{-5}$	$7.93\cdot10^{-7}$
3.0	$1.67\cdot10^{-3}$	$1.30\cdot10^{-4}$	$3.89\cdot10^{-5}$	$1.00\cdot10^{-6}$
4.0	$1.68\cdot10^{-3}$	$1.37\cdot10^{-4}$	$3.95\cdot10^{-5}$	$1.17\cdot10^{-6}$
5.0	$1.71\cdot10^{-3}$	$1.44\cdot10^{-4}$	$4.01\cdot10^{-5}$	$1.31\cdot10^{-6}$

[1] C_{ph} is the concentration of HML in the organic phase of the distribution system at time T;

[2] C_{ac} is the concentration of HML in the acceptor cell at time T;

References

1. Bhesaniya, K.D.; Nandha, K.; Baluja, S. Measurement, correlation and dissolution thermodynamics of biological active chalcone in organic solvents at different temperatures. *J. Chem. Thermodyn.* **2014**, *74*, 32–38. https://doi.org/10.1016/j.jct.2014.03.026.

2. Just, S.; Sievert, F.; Thommes, M.; Breitkreutz, J. Improved group contribution parameter set for the application of solubility parameters to melt extrusion. *Eur J Pharm Biopharm*. **2013**, *85*, 1191–1199. https://doi.org/10.1016/j.ejpb.2013.04.006.
3. Hansen, C.M. Hansen solubility parameters: A user's handbook. CRC Press
Taylor & Francis Group, 2007, http://www.taylorandfrancis.com, http://www.crcpress.com.
